# Genetic diversity of common bean (*Phaseolus vulgaris* L.) ecotypes from Pakistan using Simple Sequence Repeats

**DOI:** 10.1016/j.sjbs.2022.103300

**Published:** 2022-04-22

**Authors:** Sammyia Jannat, Asad Hussain Shah, Mahmood ul Hassan, Ahmad Sher, Sajid Fiaz, Basem H. Elesawy, Khadiga Ahmed Ismail, Ahmad El Askary, Amal F. Gharib, Abdul Qayyum

**Affiliations:** a Department of Biotechnology, University of Kotli, Azad Jammu and Kashmir, Pakistan; b Department of Plant Breeding and Genetics, PMAS-Arid Agriculture University Rawalpindi, Rawalpindi 46300, Pakistan; c College of Agriculture, Bahauddin Zakariya University, Bahadur Sub-Campus, Layyah 31200, Pakistan; d Department of Plant Breeding and Genetics, The University of Haripur, Haripur 22620 Pakistan; e Department of Pathology, College of Medicine, Taif University, P.O. Box 11099, Taif 21944, Saudi Arabia; f Department of Clinical Laboratory Sciences, College of Applied Medical Sciences, Taif University, P.O. Box 11099, Taif 21944, Saudi Arabia; g Department of Agronomy, The University of Haripur, Haripur 22620 Pakistan

**Keywords:** Germplasm, Biodiversity, Stem anthocyanin, SSR markers, Gene pool, Breeding

## Abstract

Common bean (*Phaseolus vulgaris* L.) is a legume crop grown all over the world and is a very important food of mountain population of Pakistan for protein intake. The Western Himalayan Mountains are rich in biodiversity including unexplored landraces of the common bean crop. Unfortunately, very little attention has been given to this valuable crop in Pakistan, and it is being exported, majorly from Ethiopia, to meet the country’s requirements. The exploitation, utilization, preservation and multiplication of existing germplasm within the area are very important for sustainable production of the crop and enhancing the nutrition value for the local community in mountain regions. A research study was conducted for evaluation of biological diversity of common bean landraces from Azad Kashmir and Northern areas of Pakistan using morpho-physiological and molecular markers. Thirty-five common bean ecotypes along with one check variety were collected from different altitudes of Azad Kashmir and Northern Pakistan and screened for biological diversity. Morphological characterization revealed high genetic diversity in parameters including stem anthocyanin, growth type, days to flowering, pods/plant and 100 seeds weight. Genomic characterization using SSR markers, for allelic diversity evaluation among germplasm, also provided diverse profile with 83.3% polymorphism in banding pattern. The bulk of gene pool diversity evaluated within bean landraces may help to initiate breeding program for common bean improvement.

## Highlights

• An outlook for the diversity of common beans germplasm in Azad Kashmir and Gilgit valley.
• An insight in the phenotypic diversity assisted with the SSR marker based diversity for ecotypes selection.
• A screened gene pool, to initiate hybridization program for crop improvement.

## 1 Introduction

According to the classification of United Nations, Pakistan comes under the category of lower middle income nations although the mountain areas are under least developed category (Shah et al., 2009). Concomitant with this, spectrum of poverty and eroded resource base is the dilemma of mountain people. Countries like Pakistan are facing serious problems of malnutrition due to low protein in their population diet (Khaliq et al., 2021). However, according to the 2018 national nutrition survey, 36.9% of the population is food insecure (UNICEF, 2021). Common bean (*Phaseolus vulgaris* L.) is nutritionally very rich annual legume crop, taken as green and dry beans for major plant protein source for rural and urban population throughout world (Atilla et al., 2010).

Common bean is major crop of the mountain population of Pakistan after wheat and maize for cheaper protein intake. In the area farming communities manage to cultivate common bean landraces through intercropping with maize and use it for crop rotation (Danish et al., 2002). Common bean is major and inexpensive source of protein comprising essential amino acids like lysine in contrast to animal protein. It is also rich in carbohydrates, dietry fibers, minerals, anioxidants like polyphenols and vitamins (Broughton et al., 2003).

Common bean crop has tremendous genetic resources with promising future in Pakistan and AJ&K (Hayat et al., 2014). Despite of its importance and potential, no significant research work has been reported for crop improvement. Major factors responsible for low yield in mountain areas of Pakistan are decrease in soil fertility, steep slopes, erosion, rapid population growth, strong competition to cereals and cash crops, lack of effective research programs and its improper channelization and unavailability of commercially grown varieties to farming communities. Despite of its low production in Pakistan its consumption is increasing among mountain communities for protein intake (Amanullah et al., 2006; Kumar et al., 2008). Still no well-defined variety is developed and provided to the farmers by exploiting the natural diversity. Traditionally farmers rely on the seed conserved of their own for its cultivation (Amanullah and Muhammad, 2011).

Landraces management and development of high yielding, good quality, disease resistant and environmentally adaptable cultivar is the only remedy of the prevailing problems. It is achievable by exploring bulk of biodiversity within area and using these diversified germplasm resources which are already adaptable to local conditions for common bean improvement program (Amanullah et al., 2006, Villa et al., 2006).

Presence of adequate and diverse germplasm (landraces) within Northern areas and Azad Kashmir may help to explore diverse genepool of the crop. Characterization and utilization of the natural diversity for common bean improvement will help to diminish malnourishment problems in mountain population and diversify the conventional agricultural system to create more economic opportunities for sustainable livelihoods. In this study common bean ecotypes from different pockets of Azad Jammu and Kashmir and Gilgit Baltistan were collected and screened for morphological diversity and allelic differences based on SSR markers.

## 2 Materials and methods

Thirty-five (35) ecotypes/landraces from different locations of Azad Kashmir and Northern areas of Pakistan were collected and utilized for research program with one check variety from CIAT (International Centre for Tropical Agriculture) shown in Table 1. Soil beds were prepared using standard agronomic practices and recommended fertilizer doses. Seeds were sown in RCBD (Randomized complete Block design) for morphological screening with row to row distance 60 cm while plant to plant distance of 20 cm.

**Table 1:** Common bean (*Phaseolus vulgaris* L.) ecotypes collected from different regions of Pakistan.

**Sr. No.**	**Ecotypes**	**Source**	**Seed coat color**	**Sr. No.**	**Ecotypes**	**Source**	**Seed coat color**
1	E1	Neelum	Light brown and red	19	E19	Ghizar	Green
2	E2	Neelum	Light brown and black	20	E20	Lipa	Light brown n black
3	E3	Neelum	Red	21	E21	Khursheed Abad	Red Striped
4	E4	Neelum	Red and light brown	22	E22	Khursheed Abad	Light brown and red
5	E5	Banjosa	Red	23	E23	Ghizar	Green striped
6	E6	Neelum	Light brown yellow	24	E24	Khursheed Abad	Black
7	E7	Lipa	Black	25	E25	Dhamni	Black n light brown
8	E8	Forward Kahuta	Red	26	E26	Ghizar	Yellow
9	E9	Neelum	Black	27	E27	Lipa	Light brown
10	E10	H.Kot	Light brown and red	28	E28	Ghizar	Pink
11	E11	Lipa	Black	29	E29	Ghizar	Light brown and red
12	E12	Khursheed Abad	Long red	30	E30	Ghizar	Red
13	E13	Forward Kahuta	Red striped	31	E31	Lipa	Red
14	E14	Khursheed Abad	Light brown	32	E32	Lipa	Light brown and red
15	E15	Khursheed Abad	Light brown with black	33	E33	Lipa	Yellow
16	E16	Forward Kahuta	Pinkish red striped	34	E34	Athmuqaam	Light brown and black
17	E17	Khursheed Abad	White	35	E35	Athmuqaam	Light brown and red
18	E18	Khursheed Abad	Red	36	Check	CIAT	Red

### 2.1 Morphological studies

A field experiment was conducted during the planting season, with thirty-five landraces and one check variety from CIAT of common bean in Rawalakot, Azad Jammu and Kashmir (Latitude 33°51′32.18″N, Longitude 73° 45′34.93″E and an Elevation of 5374 feet). The morphological studies were conducted under the following parameters described by common bean descriptor provided by NARC Islamabad. Qualitative attributes including leaf anthocyanin, leaf color, leaf hairiness, stem anthocyanin, number of branches, growth type, flower bud size, flower bud shape, flower keel color were observed. Similarly quantitative attributes including sowing date, days to germination, germination percentage, days to flowering, days to pods formation, number of pods per plant, number of seeds per pod, days to maturity and 100 seed weight were observed to evaluate genetic diversity among thirty-six common bean ecotypes.

### 2.2 Molecular analysis

#### 2.2.1 Genomic DNA extraction

Genomic DNA was isolated using the procedure described by Doyle and Doyle (1987). DNA quality was checked by running the genomic DNA sample on 0.8% Agarose gel.

#### 2.2.2 Conditions optimized for SSR analysis

Simple Sequence Repeats (SSR) primers, shown in Table 2, for genetic diversity in common beans were used in PCR reaction for all ecotypes. For SSR analysis concentration of genomic DNA, 10X PCR buffer, MgCl_2_, dNTP's, primers and Taq polymerase were optimized for 20 µ*l* (1X) reaction mixture. Samples were run on agarose gel and then the DNA bands were visualized on UV-transilluminator and gels was photographed using gel documentation system.

**Table 2:** Simple Sequence Repeats (SSR) primer sequences (reverse and forward primer) for diversity evaluation.

**Sr. No.**	**Primer**	**Primer Sequence**	**Sr. No.**	**Primer**	**Primer Sequence**
1	(ATGC)4-A	TGCCACCACAGCTTTCTCCTC	8	(AT)8-B	TCACGTTATCACCAGCATCA
2	(ATGC)4-B	TATGAGAGAACGGTTGGCAG	9	(AG)8-A	TTGATGACGTGGATGCATTC
3	(GGC)5-A	CTGAAGCCCGAATCTTGCGA	10	(AG)8-B	AAAGGGCTAGGGAGAGTAAGTTGC
4	(GGC)5-B	CGCGAGAGGTGAACGAGTG	11	(CCCT)3-A	CACCAATGTCTCCGGCGCA
5	(TA)22-A	GGGAGGGTAGGGAAGCAGTG	12	(CCCT)3-B	CGGTTGCCGTCGAATGTGAT
6	(TAA)22-B	GCGAACCACGTTCATGAATGA	13	(AT)9-A	AGTCGCCATAGTTGAAATTTAGGTG
7	(AT)8-A	GTTTCTTCCTTATGGTTAGG	14	(AT)9-B	CTTATTAAAACGTGAGCATATGTATCATTC

### 2.3 Statistical analysis

Multivariate analysis including factor analysis and cluster was carried out for data of morphological parameters with the help of window based computer softwares Statistica 5.0. Dendrogram was constructed by the UPGMA for qualitative analysis and molecular analysis while results for quantitative parameters were analyzed by ward's method using squared Euclidian distance.

## 3 Results

### 3.1 Morphological diversity

#### 3.1.1 Cluster analysis

##### 3.1.1.1 Hierarchical clustering (Qualitative attributes)

**Fig. 1:**
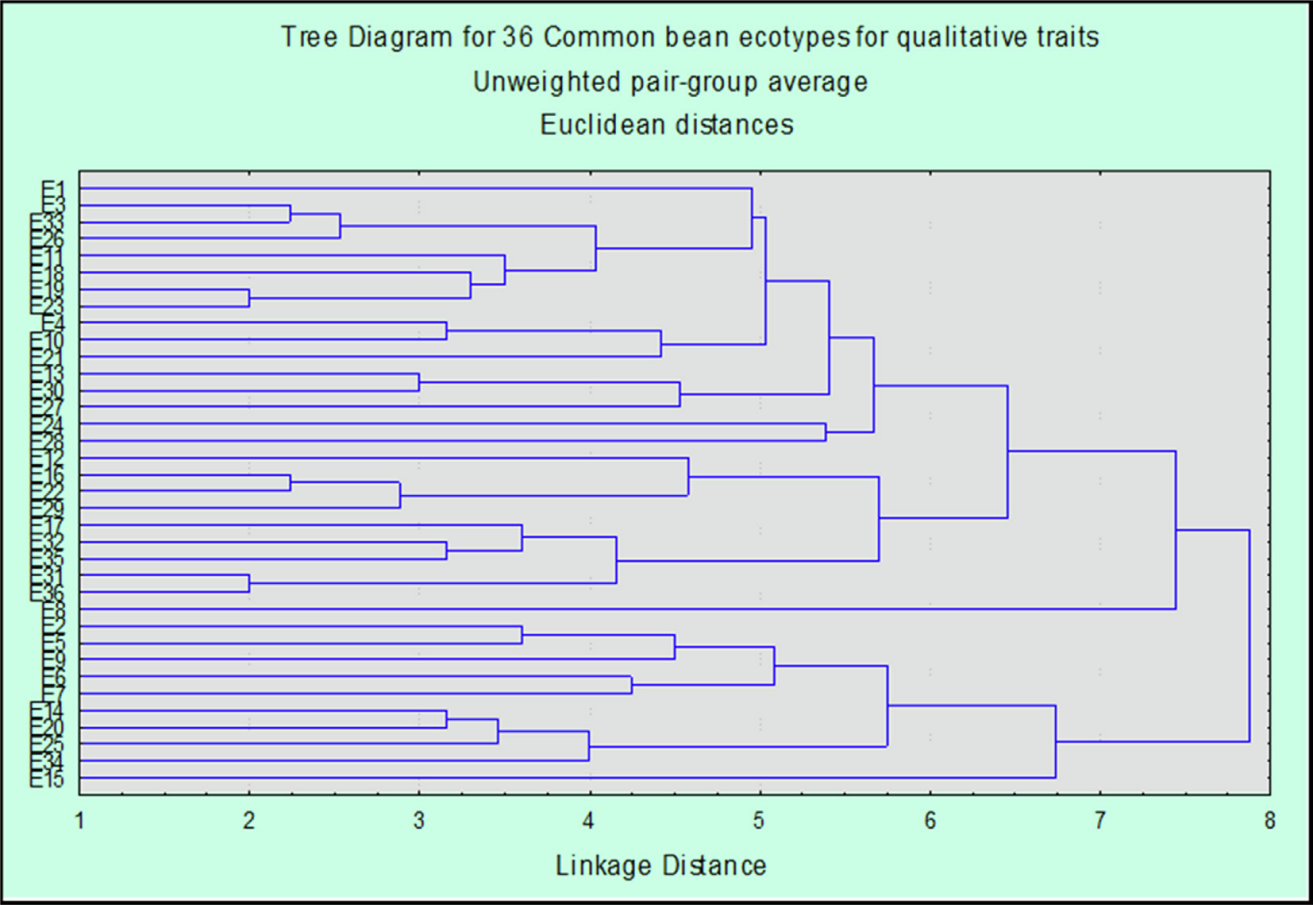
Dendrogram based on average linkage distance for qualitative traits for 36 common bean ecotypes.

The tree diagram showed inheritance pattern and relationship among thirty-six common bean ecotypes based on different qualitative characters (Fig. 1). According to qualitative characters the gene pool was categorized in two main clusters which were further divided in sub clusters based on similarities and differences. Qualitative parameters were depicting very interesting picture of variation as both main clusters showed two most diverse and variant ecotypes at larger linkage distance. Sub clusters also showed different variants revealing that there is greater variation in genotypes based on the qualitative characters. At linkage distance of six units the ecotypes were grouped in five logical clusters. Ecotype E15 in first cluster showed very diverge behavior in plants morphology. In sub cluster IIa out of four ecotypes, two were variants, E34 and E25 which showed diverse pattern. Sub cluster IIb was further classified into two more clusters with ecotypes E7, E6, E9, E5 and E2. Ecotype E9 was variant in this sub cluster while E5 and E2 were geographically from different localities and with different seed coat color were placed at same linkage group in the map. Moving above the tree diagram E8 was showing the most diverse pattern in its qualitative traits. It was found variant with diverse pattern in its morphological characters. Next cluster contained nine ecotypes grouped again in two sub clusters, IIIa and IIIb. Cluster IIIa was further comprised of two groups one containing two genotypes and one comprised of three. Some ecotypes with different phenotypic characters of seeds were clustered in same groups at same linkage distance within tree diagram. While E17 was an outlier in this group. Sub cluster IIIb is comprised of four ecotypes, E29, E22, E16 and E12, ecotype E29 was variant at larger linkage distance in map in this sub cluster. Next to this group, there was another variant in the same sub cluster named E12. Last cluster, which incised at linkage distance of six unit, was the cluster with more number of ecotypes. It contains sixteen ecotypes including, E28, E24, E27, E30, E13, E21, E10, E4, E23, E19, E18, E11, E26, E33, E3 and E1. Cluster IV was further classified in two sub clusters IVa and IVb. Sub cluster IVa contained two ecotypes E28 and E24. Sub cluster IVb comprised of 14 ecotypes in further sub clusters ecotype E27 was outlier in this group. There were two more variants in the same group *i.e.* E18 and E11 and in next group of sub cluster IVb, E26 was diverse. Ecotype E1 was showing the diverse pattern among these fourteen ecotypes in its qualitative characters. Dendrogram revealed a greater differentiation between ecotype E1 and E15. These two ecotypes were quite apart from each other in tree diagram exposing diverse ancestry in their phylogenetic behavior. From the results, based on the similarities and differences, clustering of the ecotypes showed that despite of genetic pattern and geographic effects, seed coat color and pattern of the genotypes have great impact on the development of qualitative traits of the plants in common bean specie.

##### 3.1.1.2 Analysis of variance

Analysis of variance in qualitative attributes is displayed in the Table 3. Table interprets the significance level of all variables for their F values. Significance level was checked on P ≤ 0.1. Stem anthocyanin, leaf hairiness and flower color showed highly significant variability. While leaf anthocyanin and flower bud size showed significant diversity. All other qualitative parameters displayed non-significant results for variance analysis.

**Table 3:** Analysis of Variance (ANOVA) for qualitative attributes of common bean (*Phaseolus vulgaris*).

	Between SS	df	Within SS	df	F	signif. p
Leaf anthocyanin	2.64	4	5.36	31	3.82	0.01*
Leaf color	12.85	4	46.15	31	2.16	0.10 ^ns^
Leaf hairiness	79.58	4	51.31	31	12.02	0.000005^**^
Stem anthocyanin	186.18	4	42.57	31	33.89	0^**^
No. of branches	2.32	4	36.23	31	0.50	0.74 ^ns^
Growth type	5.18	4	54.04	31	0.74	0.57 ^ns^
Flower bud shape	3.09	4	32.55	31	0.74	0.57 ^ns^
Flower bud size	11.71	4	32.18	31	2.82	0.04*
Flower colour	160.49	4	67.73	31	18.36	0^**^

##### 3.1.1.3 Cluster means

**Fig. 2:**
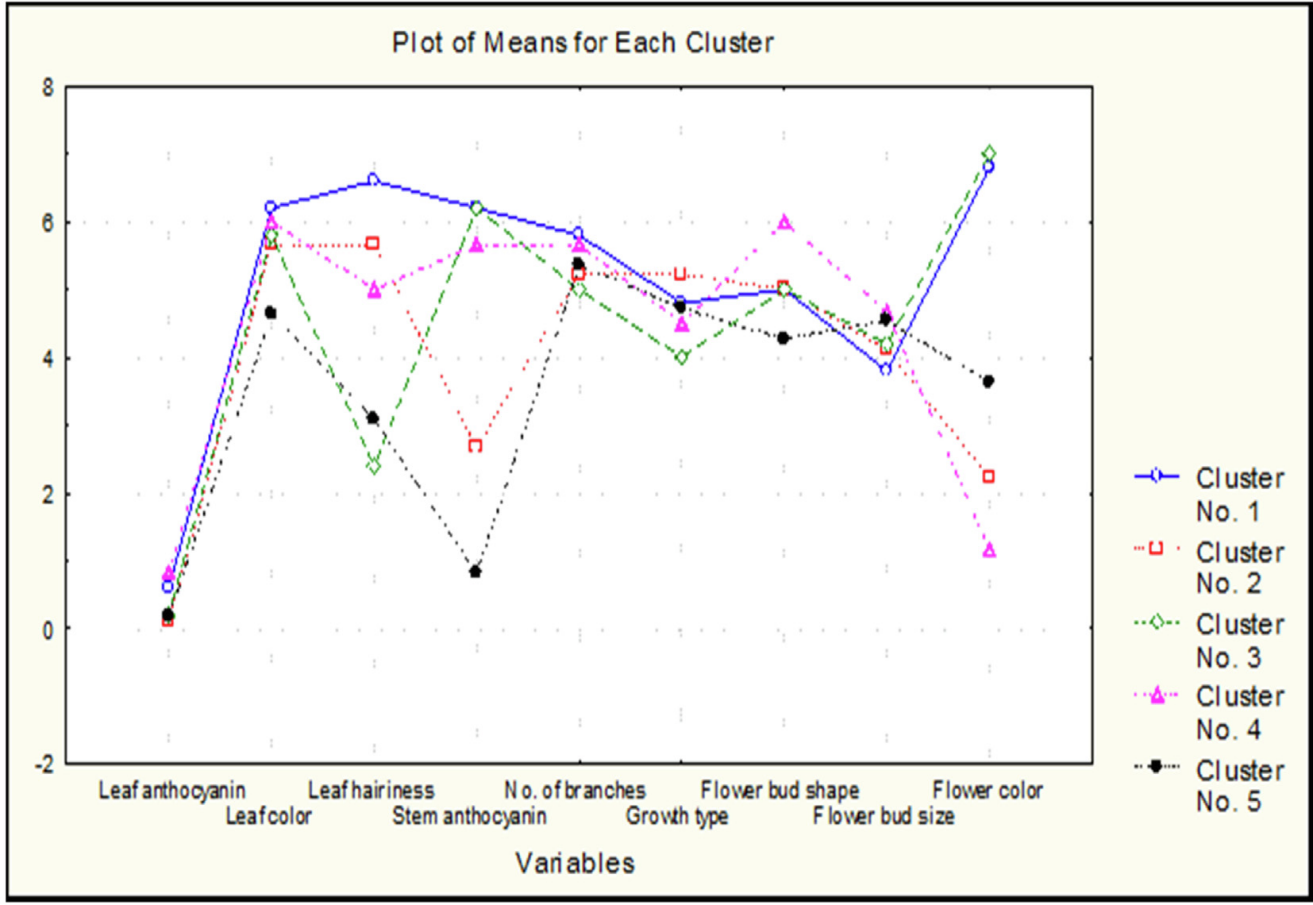
Clusters mean for qualitative characters in 36 common bean ecotypes.

Mean values of each variable in five clusters is displayed in the Fig. 2. The figure represents stem anthocyanin in cluster 5 as more diverse variable. More diversity was found in the members of all five clusters for stem anthocyanin, leaf hairiness and flower color.

##### 3.1.1.4 Factor and principle component analysis

Principle component analysis for nine qualitative characters of various common bean ecotypes is shown in the Table 4. Four factors with Eigen value >1 were extracted as shown in Table 2. Maximum eigen value was observed in factor 1 *i.e.* 2.05. These major four components contribute 69.12% variability of total variation. Factors 1 explained 22.74%, factor 2 revealed 17.79%, factor 3 explained 15.76% and factor 4 elucidated 12.82% of total variance.

**Table 4:** Principal Components (PCs) for qualitative attributes in 36 common bean ecotypes.

Eigen values				
	PC 1	PC 2	PC 3	PC 4
Eigen value	2.05	1.60	1.42	1.15
% total variance	22.74	17.80	15.76	12.82
Comulative Eigen value	2.05	3.65	5.07	6.22
Comulative %age	22.74	40.53	56.29	69.11

### 3.2 Cluster analysis (Quantitative attributes)

#### 3.2.1 Hierarchical cluster

**Fig. 3:**
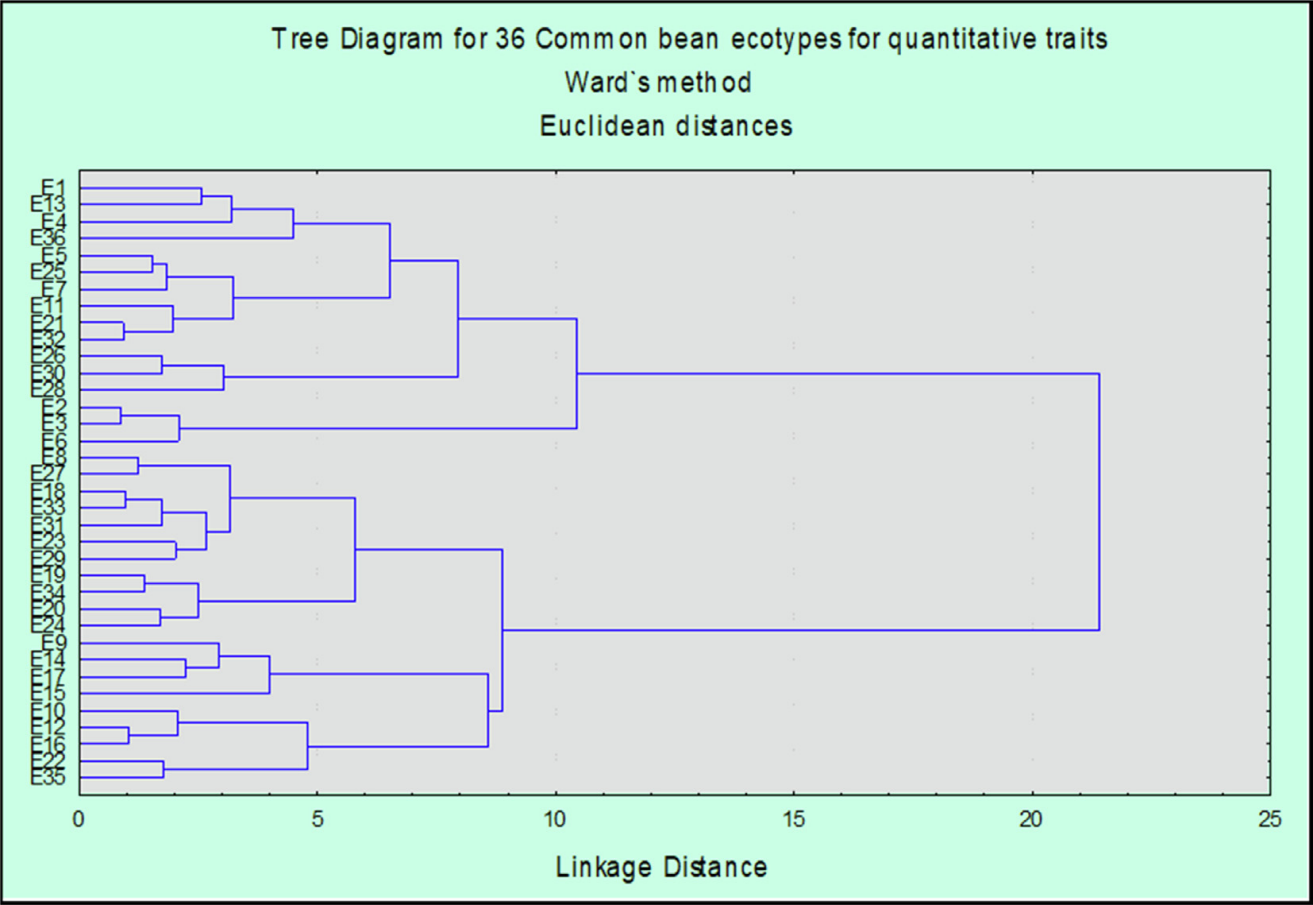
Dendrogram based on average linkage distance for quantitative traits of 36 common bean ecotypes.

The cluster diagram of 36 common bean ecotypes obased on different quantitative characters is displayed in Fig. 3. The dendrogram indicated a linkage map amongst the 36 ecotypes of common bean specie. All 36 ecotypes were grouped in two main clusters based on their quantitative characters. At linkage distance of 8 units, dendrogram was grouped in six clusters. At this incision point moving from bottom to top of the tree diagram first cluster named as cluster I contained five ecotypes E35, E22, E16, E12, E10 representing the true relationship in the phenolgy of these ecotypes, E10 was variant in cluster Ib. Cluster II has further sub clusters and contains four ecotypes *i.e.* E15, E17, E14 and E9 and E15 was outlier in this group. Ecotype E9 with black seed cover was at larger linkage distance as compared to E17 and E14 of the same cluster. Next cluster III grouped 11 ecotypes with two sub clusters IIIa and IIIb included E24, E20, E34, E19 in sub cluster IIIa and E29, E23, E31, E33, E18, E27 and E8 in sub cluster IIIb, the red beans E31 was variant. In the above tree diagram, cluster IV put on view of only three genotypes in it including E6, E3 and E2. Ecotype E6 was variant in this group. This cluster presented a clear picture of influence of geographical back round on the quantitative performance of common bean ecotypes. Cluster V exhibited only three genotypes with one E28 a variant and E30 and E26 at same linkage distance in this group. The last cluster contained the most diverse ecotypes from the studied gene pool. Cluster VI was comprised of two sub clusters VIa and VIb, sub cluster VIa contains E32, E21, E11, E7, E25 and E5 while VIb is comprised of four genotypes consist of E36, E4, E13 and E1, E11 was a variant in sub cluster. In sub cluster VIb E36 was check variety with diverse quantitative characters and red seed coat color. The dendrogram revealed that E36 is a variant, positioning at larger linkage distance, among 36 studied genotypes. This genotype was quite different in its germination, growth, yield and maturity pattern. While E4, E13 and E1 were closely related to the check variety in their quantitative traits. E15 followed by E4 were also showing variation in their quantitative parameters among all other genotype. Similarly greater phylogenetic distance was shown by ecotypes E1 and E35, both ecotypes showed distinct heritage pattern in the cluster and revealed significant divergence from the common ancestors.

#### 3.2.2 Clusters means

**Fig. 4:**
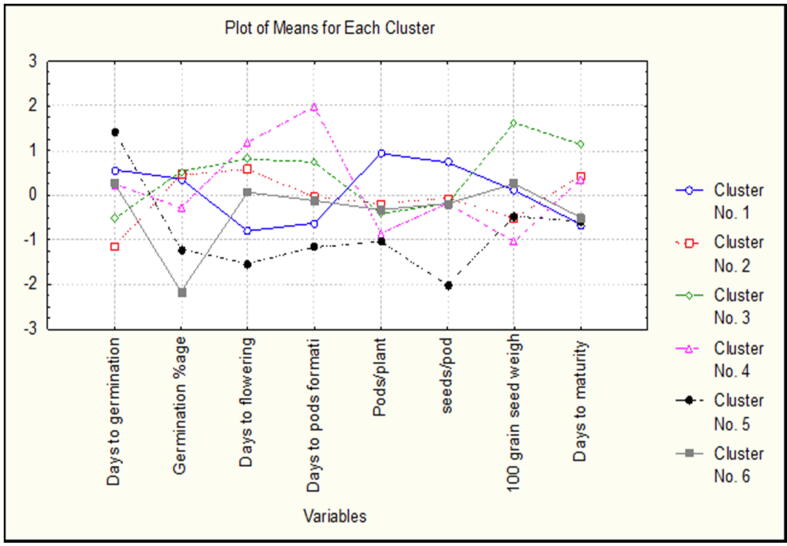
Cluster mean for quantitative attributes in 36 common bean ecotypes.

Cluster mean of 36 common bean ecotypes for their quantitative attributes were displayed in Fig. 4. Mean values for pods/plant and seeds/plant were higher in cluster I *i.e.* 0.94 and 0.73. In cluster II mean values for days to flowering is more 0.59 followed by germination percentage 0.46. Parameter 100 grains weight has higher mean value *i.e.* 1.62 in cluster III followed by days to maturity 1.14. Members of cluster IV have higher value for days to pods formation 1.98 and days to flowering 1.18. Mean value for days to germination (1.44) was higher in members of cluster V. Similarly ecotypes in cluster VI has higher mean value for germination percentage *i.e.* 0.28.

#### 3.2.3 Analysis of variance

Analysis of variance in quantitative variables is displayed in the Table 5. Table revealed the significance level of all parameters based on the F values. Significance level is checked on P ≤ 0.1. all the parameters were showing highly significant results in analysis of variance with their p values less than 0.1. Significant diversity was reported by Nkhata et al. (2020) in different morphological traits.

**Table 5:** Analysis of variance for quantitative attributes of common bean.

	Between SS	df	Within SS	df	F	signif. p
Days to germination	23.78	5	11.22	30	12.71	0.000001^**^
Germination %age	23.58	5	11.42	30	12.39	0.000001^**^
Days to flowering	26.69	5	8.31	30	19.27	0^**^
Days to pods formation	27.29	5	7.71	30	21.25	0^**^
Pods /plant	18.14	5	16.86	30	6.46	0.00035^**^
Seeds/pod	19.04	5	15.96	30	7.16	0.0002^**^
100 grains weight	20.82	5	14.18	30	8.811	0.000031^**^
Days to maturity	15.66	5	19.34	30	4.86	0.002261^**^

#### 3.2.4 Principal component analysis of quantitative attributes

Principal component analysis for some of the quantitative attributes in various common bean ecotypes was shown in Table 6. Three components with Eigen value >1 were extracted. Factor 1 shows maximum Eigen value of 2.81 with 35.10% of the total variance amongst the studied ecotypes. Factor 2 explained 24.61% and factor 3 elucidated 12.73% of the total variance explained. All three factors contributed 72.45% variability among the studied ecotypes.

**Table 6:** Principal Components (PCs) for quantitative attributes in 36 common bean ecotypes.

Eigen values			
	PC 1	PC 2	PC 3
Eigen value	2.81	1.97	1.02
%age variance	35.10	24.61	12.74
Cumulative Eigen value	2.81	4.78	5.80
Cumulative %age	35.10	59.71	72.4

#### 3.2.5 SSR primers amplification

**Fig. 5:**
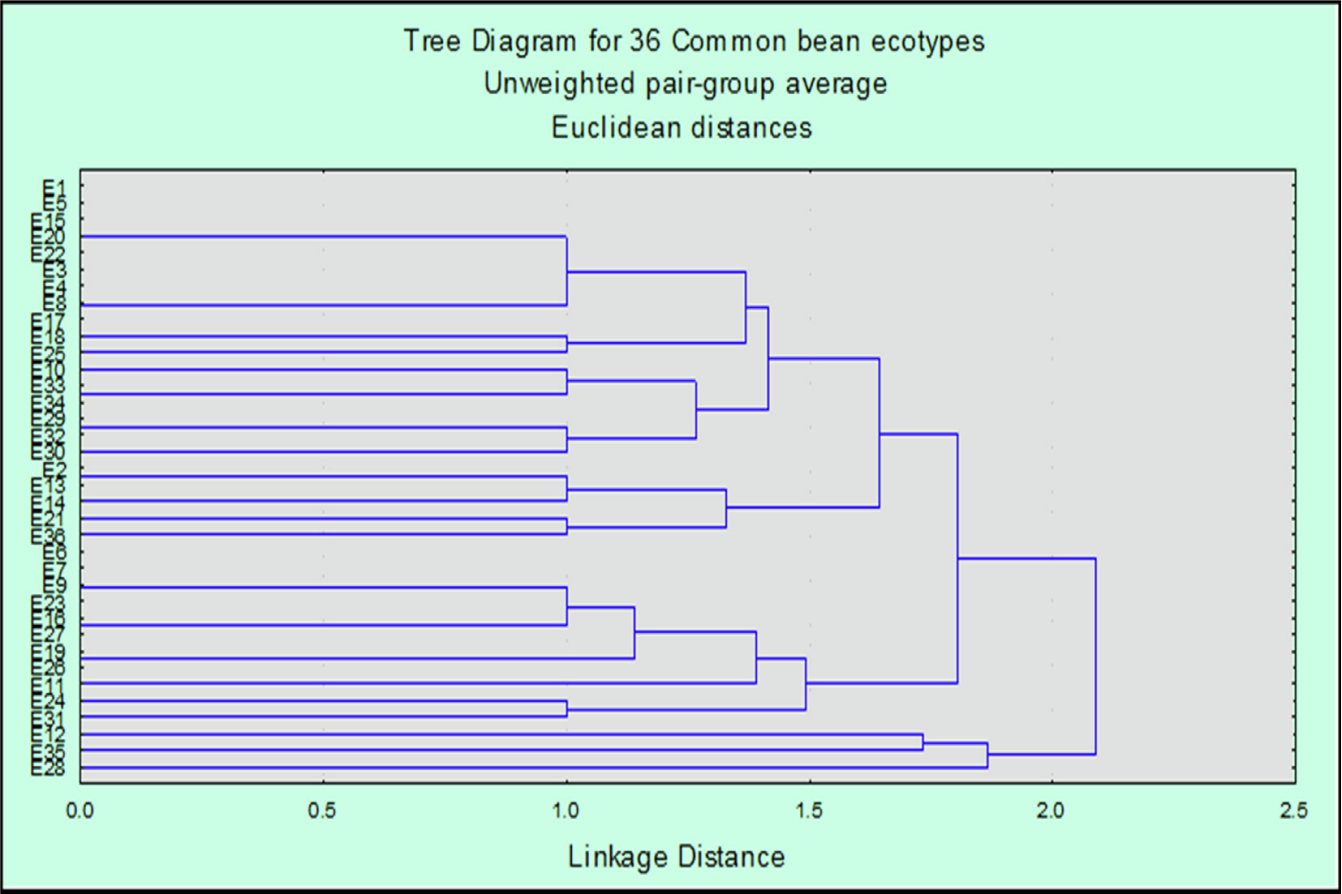
Dendrogram of 36 common bean ecotypes of common bean for SSR markers.

Cluster diagram of 36 common bean ecotypes for their diversity evaluation by using molecular markers has been shown in Fig. 5. Total seven primers were used for diversity evaluation, out of which four showed polymorphic bands while three are of monomorphic nature. The UPGMA dendrogram based on genetic distances among populations showed very clear picture of specie’s evolutionary pattern in correlation and variability. Diversity screening of 36 common bean ecotypes by using SSR primers characterized all ecotypes in two main clusters, cluster I and cluster II. Ecotypes at zero linkage distance in tree diagram were also represented and showed least diversity among all ecotypes. Cluster I contained three ecotypes with genetically most diverse ecotype E28 and other two in same cluster at same linkage distance, but all these three ecotypes were at larger linkage distance in tree diagram. Cluster II is further sub clustered as IIa and IIb. Group IIa was comprised of six ecotypes while IIb exhibited twelve ecotypes based on their polymorphism pattern, E11 and E19 were outliers at larger distance in e diagram with more genetic variations. These both were diverse enough in sub cluster IIa. All ecotypes in cluster IIb are from different geographical zones with difference in their seed coat colors. Ecotype E20 and E28 were far away from each other showing least phylogenetic relation.

This clustering pattern revealed the similarity and difference index of all 36 ecotypes of common bean on their genetic makeup. Total 12 bands were found in all 36 common bean ecotypes, out of which only two showed monomorphic behavior, while other ten were polymorphic bands. SSR study revealed 83.3% polymorphism in banding pattern of 36 common bean ecotypes. These results indicated presence of higher amount of diversity in the collected landraces. Significant variation in the germplasm is confirmed by using these molecular markers.

## 4 Discussion

Cluster analysis along with PCA and Factor analysis was performed for identification of genetic diversity, tracing of evaluation pathway of crop, evaluation of diversity and environmental interaction due to morpho-physiological traits. The cluster analysis revealed phylogenetic relationship among 36 common bean ecotypes for qualitative as well as quantitative traits. It is clear from above results that morpho-physiological parameters hoard no marked impact of different source areas and geographic localities on their diversity pattern. Even ecotypes from different localities are clustered in same groups at same linkage distances on the tree diagram. Similarity morphological behavior of ecotypes reflected that common bean had been domesticated in different parts of the world after being transported from its center of origin. Bean landraces grown in Himalayan region possess high level of diversity for seed color, shape, size, and flavor (Choudhary et al., 2018). The investigations also unveil the fact that seed coat color and pattern has great association with the development of qualitative characters of the plant types and despite of the geography ecotypes with same seed coat color and pattern are grouped in the same clusters. It indicates that phenotypic variation is still crucial to determine the variability as morphological traits of the plants are greatly influenced by genetic factors and genotypic structure plays major role in implication the phenotypic and morphological performance of them. Due to these reasons scientists used to classify the common bean land races for seed size, shape and color (Rheenen, 1979). Similarly, Katungi et al. (2011) declared that grains color in common beans is highly important for quality attributes.

In case of quantitative parameters, influence of geographical contribution to the clustering pattern is up to certain level; otherwise quantitative attributes are independent of the effect of collection sites up to larger extent. Cluster analysis traced out the reasonable patterns of germplasm dispersion and diversification of the specie. Ecotype E8 is a variant at larger linkage distance for qualitative traits while E36 is variant in case of quantitative attributes among all studied ecotypes.

Based on the qualitative characters there is an enormous variation even among the genotypes of the same area. In case of quantitative traits, check variety showed a diverse pattern as compared to ecotypes of Azad Kashmir and Northern areas. Stem anthocyanin, leaf hairiness and growth types were observed more variant among qualitative parameters. Other variables like flower color, flower bud shape, and size, leaf color and anthocyanin and number of branches also showed high variability. All these attributes were defined as significant diversity evaluating factors in common beans (Lima et al., 2012). Shree et al. (1991) found a range of variation in flower and hypocotyls pigmentation and growth type of common beans and his results corroborates with the present facts. Similarly Morris (2008) also confirmed significant variability in anthocyanin indexes in leaves of horse gram as found in current investigation of common beans leaf anthocyanin. Diversity found in leaf color of common bean ecotypes is in accordance with Nkouannessi (2005) who reported leaf pigmentation variations in cowpea.

Variability caused by quantitative traits is more influenced by variables like days to flowering, days to pods formation, days to maturity pods/plant and 100 seeds weight. Greater variation was observed in traits like days to flowering vary from 31 to 48 days. Present range for days to flowering is in accordance with the results of Sofi et al. (2011) who reported 35–58 days to flowering in common bean landraces. As early maturity in common beans is pre-requisite for its cultivation preferences among farming communities that is why days to maturity is a crucial variable during evaluation of crop. Wide range of variation was observed for days to maturity with the range of 68–115 days. For common bean landraces variability reported in this factor agrees with the documentations of Amanullah et al. (2006) with 82–103 days range and Sofi et al. (2011) with 77–124 days range for crop maturity. The traits which are directly responsible for crop yield also showed diverse behavior *i.e.* pods/plant, seeds/pods and 100 seed weight. Similar findings were examined by Balcha (2010) for these three variables. They also exposed striking diversity in these yield generating attributes as observed in current study. It was observed that significant and positive correlation was found in yield attributes including pods per plant, seeds per pod and 100-seed weight (Assefa et al., 2019). The values for pods/plant is reported as 7–46 which is not in accordance with the pods/plant observed by Pereira et al. (2009) *i.e.* 13–19 and *i.e.* 12–27. This contrast in results may be due to the deviating behavior of specific ecotypes or that may be due to environmental differences specially fluctuations in temperature ranges. Seed/pod was also depicting a diverse pattern among 36 ecotypes ranging from 2 to 7 in numbers. These observations has great similarity with the findings of Pereira et al. (2009) *i.e.* 2–7 and Sofi et al. (2011) *i.e.* 3–6. The main parameter of yield, 100 seed weight contained values in range of 9.16–49.67 g. These findings are similar to the findings of Sofi et al. (2011) and Amanullah et al. (2006) who documented 29–60 g and 19.5–61.5 g 100 seed weight as a result of their findings respectively. Despite of diversity factor, ecotypes with high germination percentage, more 100 seeds weight and early maturity were also evaluated. Ecotype E4 was considered better among others with 75% plants germination, 45.15 g 100 seeds weight and 72 days to maturity.

The encouraging diversity exposed by different ecotypes collected from diverse localities of Azad Jammu and Kashmir and Northern areas of Pakistan for different morpho-physiological traits depicted a significant genetic variation. Set of characteristics are evaluated for defining a landrace rather than a single parameter which could further help to design a breeding program for specific crop group (Villa et al., 2006). Selection of such a diverse and genetically influential parameters may be helpful to design new breeding programs for common bean improvement and development of a new variety. In present studies the ecotypes have been pooled from assorted areas with different altitudes, moisture, environmental and geo-demographic profile.

The SSR study profile added new insights into the picture of diversity of the common bean ecotypes. SSR profiling techniques provides useful information on the level of polymorphism and diversity in common bean, showing their utility in the characterization of germplasm. Savić et al., 2021 revealed a wide range of genetic diversity based on the Neighbor-joining clustering within 118 land races using 27 SSR primers. Only four out of seven primers show polymorphic bands in their genome. Similar behavior in banding pattern was revealed by Yu et al. (2000) who found 24 polymorphic SSR primers out of 37. While Foschiani et al. (2009) reported that among 23 SSR primer pairs, only 10 were useful in common bean landraces diversity studies. Similar findings were observed in the study of Ince and Karaca (2011) for SSR primers, they reported that among 13 SSR primer pairs, only 3 produced polymorphic band in common bean landraces. This may be due to unsuitable primer sequence or PCR conditions as revealed by Akagi et al. (1996). Present SSR characterization of beans reported 83% polymorphism among all twelve bands. Similarly Maras et al., 2008, Okii et al., 2017 found different sub groups among the gene pool of common bean based on the allelic dispersion using SSR markers. More robust markers have been used to reveal a range of diversity panel in Brazilian common bean germplasm (Delfini et al., 2021). Genome wide association mapping using SSR markers revealed a wide range of genetic variation and identified marker –trait association (MTA) with yield related traits in the landraces of Jammu and Kashmir (Mir et al., 2021). Molecular assay along with morphological analysis of 36 common bean ecotypes revealed an encouraging variation pattern, which indicates that ecotypes even collected from near localities, based on their phenotypic analysis are genotypically diverse enough. Current study showed a great genetic potential of the common bean crop to initiate a breeding program for its yield improvement in the region.

## 5 Conclusion

The present study showed a significant diversity in the common bean landraces of Azad Jammu and Kashmir. Keeping in view its incredible genetic potential, a comprehensive and integrated effort to improve the existing germplasm should be initiated which may lead to diversification in conventional agricultural system and may become a profitable venture for poor farmers of the country.

## Declaration of Competing Interest

The authors declare that they have no known competing financial interests or personal relationships that could have appeared to influence the work reported in this paper.
